# Evaluation of compost, vegetable and food waste as amendments to improve the composting of NaOH/NaClO-contaminated poultry manure

**DOI:** 10.1371/journal.pone.0205112

**Published:** 2018-10-11

**Authors:** Yuting Liu, Wenxia Wang, Jianqiang Xu, Hongyu Xue, Kim Stanford, Tim A. McAllister, Weiping Xu

**Affiliations:** 1 School of Food and Environment, Dalian University of Technology, Panjin campus, Panjin, China; 2 School of Life Science and Medicine, Dalian University of Technology, Panjin campus, Panjin, China; 3 Panjin Industrial Technology Institute of Dalian University of Technology, Panjin, China; 4 Alberta Agriculture and Forestry, Agriculture Centre, Lethbridge, Alberta, Canada; 5 Agriculture and Agri-Food Canada, Lethbridge Research Centre, Lethbridge, Alberta, Canada; Estacion Experimental del Zaidin - CSIC, SPAIN

## Abstract

Regular usage of NaOH/NaClO disinfectants results in high sodium salt and alkalinity of poultry manure. This study compared three amendments: vegetable waste (V), food waste (F) and mature compost (C) for their ability to improve the composting of NaOH/NaClO-contaminated poultry manure. C compost resulted in the highest compost temperatures (*p*<0.001) and greatest reduction in OM, TC, TN and NH_4_-N (*p*<0.05). C and V composts were more efficient at lowering extractable-Na (ext-Na) and electrical conductivity (EC) than F (*p*<0.05). Maturity was primarily indicated by NH_4_-N, EC and ext-Na. Bacterial dynamics was profoundly influenced by NH_4_-N, EC and TC, with the decrease leading to discriminate genera shift from *Sinibacillus* and *Thiopseudomonas* to *Brevbacterium*, *Brachybacterium*, and *Microbacterium*. These findings suggest that mature compost was more desirable amendment than vegetable and food waste in the composting of NaOH/NaClO-contaminated poultry manure, and the decrease of ext-Na indicated compost maturity but did not influence bacterial dynamics.

## Introduction

Hygiene in animal production plays an important role in both farm management and food safety. Cleaning and disinfection of poultry houses is crucial to manage poultry farm hygiene [1]. However, after use, the disinfectants may remain in the poultry manure making it less biodegradable and more difficult to compost [2]. Common disinfectants contain bases(e.g. sodium hydroxide), acids (e.g. citric acid) oxidizers (e.g. sodium hypochlorite), quaternary ammonium compounds (QAC, e.g. alkyldimethylbenzylammonium chloride), and aldehydes (e.g. glutaraldehyde) [3,4]. Many commercial disinfectants, such as QACs and sodium hypochlorite, were proved to be highly efficient in the inactivation of bacterial isolates [4], however, their potential inhibition effect on manure composting was largely unknown and less studied. Since NaOH and/or NaClO are widely used sanitizers due to their wide availability and low cost, it is important to develop and evaluate methods to compost poultry manure containing NaOH/NaClO sanitizers, in order to enable their neutralization and make them suitable as an organic fertilizer.

Previous studies on the composting of poultry manure containing NaOH/NaClO sanitizers are rare. We found a local poultry farm using NaOH/NaClO twice weekly for routine cleaning, and the poultry manure was determined for approximately pH 8.5–8.9, extractable-Na 0.94–1.02% and electrical conductivity (EC) 26.3–34.9 mS/cm (unpublished data), which were much higher than the standard poultry manure of pH 5.8–7.7, extractable-Na 0.45–0.55% and EC 7.0–11.8 mS/cm [5–7]. Since NaClO can be transformed to NaCl after exposure to air, poultry manure containing NaOH/NaClO compared to standard poultry manure is highly alkaline, high in sodium salt and exhibits high electrical conductivity. For both high alkalinity and sodium may inhibit microbial activity and lower the value of compost as a fertilizer [6,8], it is essential to establish practical methods for composting NaOH/NaClO-contaminated poultry manure and assess the soluble Na immobilization ability among methods. Vegetable and food waste are widely available organic wastes in the rural site, which could be used as compost amendments, in order to introduce more microbial diversity, dilute manure sodium content, and dispose all the organic waste at the same time. Mature compost could be another option for compost amendment, which may dilute manure sodium content and introduce compost favorable microorganisms simultaneously. However, amendment effects on composting NaOH/NaClO-contaminated poultry manure is still unknown, which requires further evaluation on both the composting process and microbial diversity aspects.

Composting is a biological process driven by endogenous or inoculated microorganisms and influenced by the physicochemical properties of the decomposing organic matter [9,10]. Using modern next-generation sequencing technology to characterize the bacterial community is an efficient way to understand the endogenous bacterial composition and the potential environmental impact on bacterial dynamics [11–13]. The bacterial communities within chicken carcass/manure, cow manure, pig manure and green waste compost have been widely investigated with NGS, while Proteobacteria, Bacteroidetes, Firmicutes, Actinobacteria, and Chloroflexi phyla were found associated with the degradation of organic carbon and nitrogen [14–17]. The bacterial community of NaOH/NaClO-contaminated poultry manure compost is interesting to be studied by NGS and compared with other manure composting studies, in order to characterize bacterial dynamics and identify specific and functional genera.

The objectives of the present study were i) to develop and assess the optimum composting methods for NaOH/NaClO-contaminated poultry manure by comparing effects of different amendments; ii) to characterize the variation of physiochemical properties, identify compost maturity indicators, and compare sodium immobilization ability among compost types; and iii) to illustrate bacterial composition and dynamics, compare with other composting studies, and understand the chemical impact on bacterial community changing.

## Materials and methods

### Compost construction and sampling

Poultry manure was bought from the Panjin Qinfeng poultry farm, where NaOH and/or NaClO were routinely used twice weekly for poultry house cleaning. The manure was transferred to the university composting site (40°41’17”N latitude, 122°7’20”E longitude, and 10 m altitude) and air dried for four weeks prior to composting. Three amendments, i) vegetable waste, ii) food waste and iii) mature compost were collected and their moisture and chemical properties were illustrated in the Table 1. The vegetable waste was collected from the university’s cafeteria. The food waste was cooked prior to collection from the university’s cafeteria. The mature compost was produced from horse manure compost and bought from Yingkou Lvyuan Biological Organic Fertilizer Co., Ltd, where fresh horse manure was aerobic composted in windows with weekly turnings for 3–4 wk. The moisture of mature compost was determined as approximately 43–52%, additional distilled water was added and well-mixed with the mature compost, in order to raise the moisture (>70%) of mature compost close to the other two amendments. The bulking agents were green waste consisting of fallen leaves and branch cuttings of trees and shrubs at the university. Cone shaped compost piles were built in triplicate per amendment, with a dimension of 1 m high and a diameter of 1.2 m as following.

V: poultry manure + bulking agent + vegetable waste
F: poultry manure + bulking agent + food waste
C: poultry manure + bulking agent + mature compost

**Table 1:** The chemical properties of compost components at construction.

Compost component ^a^	Moisture(%)	TC (%)	TN (%)	C/N	ext-Na (%)
NaOH/NaClO poultry manure	71	32	3.5	9	1.0
vegetable waste amendment	81	41	1.6	26	0.1
food waste amendment	87	42	8.3	5	1.1
mature compost amendment	73	28	2.4	12	0.5
green waste bulking agent	5	45	1.2	38	<0.1

^a^ All the components were determined for chemical properties in triplicate, and the average values were presented.

The wet weight ratio of poultry manure, bulking agent and amendments was approximately 4:1:1. At construction, the poultry manure was thoroughly mixed with bulking agent, and the amendments were deposited in the center of the pile, until the first turning (D7) when all compost materials were thoroughly turned and mixed. As the compost components were not fully mixed at construction and the poultry manure was the main component, the chemical properties of poultry manure were used to represent compost properties on D0.

To monitor compost temperature, duplicate K type thermocouples (Testo GmbH, Lenzkirch, Germany) were placed in the center of the pile, a temperature was recorded twice daily and averaged to obtain a daily value. Compost was turned weekly for a period of 5 weeks. Each week, samples were collected from four different locations within the pile after turning. The four samples were mixed to create a composite sample (approximately 1000 g wet weight). Triplicate composite samples for V, F and C compost were collected on D7, 14, 21, 28 and 35. And triplicate poultry manure samples were collected on D0. Samples were subjected to chemical and microbial analyses. One quarter of each sample was used to determine CO_2_ respiration rate and bacterial plate counts on the sampling day, and the remaining samples were stored at -20°C prior to further analyses.

### Chemical and microbial property analyses

Moisture content was determined by the weight difference for at least 2 h oven drying at 105°C until no change in dry weight was observed [17,18]; Organic matter (OM) was determined using dry and ground samples by weight difference after 2 h of combustion in a muffle furnace at 600°C [17,18]; pH and EC were measured as a 1:5 (w/v) water soluble extract by a P11 pH meter and K10 EC meter (Bante, Shanghai, China); total carbon (TC) and total nitrogen (TN) were determined on freeze-dried and ball-grinding samples (Ø < 0.1mm) with an Vario EL cube elemental analyzer (Elementar Analysensysteme GmbH, Hanau, Germany). Ammonia nitrogen (NH_4_-N), extractable-Na (ext-Na) and extractable-K (ext-K) were determined after liquid extraction. Compost samples were mixed 1:10 (w/v) with 1 M KCl or sodium acetate at room temperature for 1 h and centrifuged. The NH_4_-N was determined with 1 M KCl extraction using the colorimetric method as described previously [19]. The ext-Na was measured with 1 M KCl extraction and ext-K was measured with 1M sodium acetate extraction using a 361MC atomic absorption spectrophotometer (Shanghai INESA Scientific Instrument Co., Ltd., Shanghai, China) [6,7]. Moisture contents were reported as percentage of fresh weight, and other analyses reported on a dry matter basis.

Compost CO_2_ respiration rates were measured using an infrared gas analyzer system equipped with a S8-0050 CO_2_ infrared sensor (SenseAir Ab, Delsbo, Sweden) as described previously [20] with modifications. Briefly, 30 g of individual compost sample and a CO_2_ sensor were placed and sealed in a 300 mL chamber, kept at room temperature, and monitored for CO_2_ concentrations every 2 sec. The CO_2_ production in the first 30 min was recorded and defined as the CO_2_ respiration rate. The compost gemination index (GI) was determined using *Lactuca sativa* (lettuce) seeds as described previously [21]. Compost total coliforms, total bacteria and fungi were determined by 10-fold serial dilution of fresh compost samples with 1 × phosphate buffered saline (PBS) buffer (pH 7.4), followed by plating on Mac Conkey agar (Hangwei Co., Ltd., Hangzhou, China), Tryptic soy agar (Hangwei) containing 50 μg/mL cycloheximide (Sigma), and Saboraud dextrose agar (Hangwei) containing 100 μg/mL tetracycline and 100 μg/mL chloramphenicol (Sigma), respectively [18]. Plates were incubated at 37°C for 16 h, 48 h and 72 h, respectively. Poultry DNA within compost samples was quantified with real-time PCR [22], using primers [23] specific to the mitochondrial 12S rRNA gene in chickens (*Gallus*.*gallus* NC_001323). Microbial and gene quantification data were reported based on a dry matter basis.

### Bacterial community Illumina MiSeq sequencing analysis

Genomic DNA was extracted from triplicate compost samples using the QIAamp Fast DNA Stool Mini Kit (Qiagen, Hilden, Germany) following the manufacturer’s protocol. The quality of DNA was checked by 1% agarose gel electrophoresis and a UV5 nano spectrophotometer (Mettler-Toledo GmbH, Greifensee, Switzerland). Extracted DNA from triplicate compost samples was mixed in equal volumes to make a composite sample and subjected to high-throughput sequencing by Shanghai Sangon Biotech Co, Ltd. (Shanghai, China) using the Illumina MiSeq sequencing platform (Illumina Inc., CA, USA). The bacteria V6-V8 region of the 16S rRNA gene were amplified using universal primer pairs: 926F [5’- (CCCTACACGACGC TCTTCCGATCTN; barcode) AAACTYAAAKGAATTGACGG-3’] and 1392R [5’- (GACTGGAGTTCC TTGGCACCCGAGAA TTCCA; barcode) ACGGGCGGTGTGTRC-3’). The PCR mixture contained (final concentrations) 3 μL extracted DNA, 0.3 μM each primer (10 μM), 15 μL 2×Taq master Mix (P111-03, Vazyme Biotech Co., Ltd, Nanjing, China) in a final volume of 30 μL. The PCR amplification was carried out on a T100^TM^ Thermal Cycler (Bio-RAD, CA, USA) using conditions of 94°C for 3 min; 5 cycles of 94°C for 30 sec, 45°C for 20 sec, 65°C for 30 sec; and 20 cycles of 94°C for 20 sec, 55°C for 20 sec, 72°C for 30 sec; with a final extension at 72°C for 5 min. PCR products were purified and then applied to the MiSeq Genome Sequencer (Illumina) according to manufacturer’s standard protocols.

To analyze MiSeq sequencing results, the raw sequence reads were analyzed sequentially by truncating the 3’ adapter sequences (Cutadapt v.1.2.1), merging paired sequences (PEAR v.0.9.6), selecting individual sample sequences based on eight nucleotide barcodes, discarding raw reads shorter than 200 nucleotides, and discarding raw reads with an average quality score <20 (Prinseq v.0.20.4). Chimeric sequences were identified and removed using Usearch (v. 5.2.236). High-quality sequences were selected based on sequence length, quality, primer and barcode tag. Only sequences containing an overlap longer than 10 bp were assembled according to their overlap sequences. The unique sequence set was classified into operational taxonomic units (OTUs) under a threshold of 97% identity using the Usearch. The taxonomy of each 16S rRNA gene sequence was classified against the RDP 16S rRNA database (http://rdp.cme.msu.edu/misc/resources.jsp) with a confidence threshold of 80% using RDP classifier (v.2.12) software, and the genus with highest confidential score was defined as the taxonomy. The MiSeq sequencing data have been deposited in the NCBI Sequence Read Archive (http://www.ncbi.nlm.nih.gov/sra) under accession number SRP158897 and BioProject number PRJNA487972.

### Statistical analyses

All the statistical analyses were carried out in R 3.4.1 [24]. Differences in the physicochemical and microbial properties of compost types and time were assessed by two-way analysis of variance (ANOVA) using *car* [25] and *lsmean* [26] packages. Correlations among the physicochemical and microbial properties were determined using *cor* and *panel* function. Differences in bacterial communities among compost types were assessed by the analysis of similarities method (ANOSIM) using *vegan* package [27]. Significance was defined as *p<0*.*05*.

Classification analysis of compost samples based on either chemical property or bacterial diversity was carried out using non-metric multidimensional scaling (NMDS) analysis with standardized data provided by *decostand* function in the vegan package [27]. NMDS analysis is an ordination method that plots dissimilar objects far apart in ordination space and similar objects close to one another, and plots deviances according to their contributions to the clustering, with deviances contributing more to the classification plotted further away from the ordination center. Interaction analyses of compost chemical property and microbial diversity were carried out by constrained classification methods of multivariate regression tree analysis (MRT) and redundancy analysis (RDA). Both MRT and RDA were performed using chemical properties as explanation deviances and bacterial compositions as response deviances. MRT produces a clustering tree with samples classified in tree leaves, chemical properties explained tree branches, and discriminate bacterial species identified in tree nodes and leaves. RDT produces ordination results with samples, chemical properties and bacterial species plotting in one ordination space, and is interpreted generally similar to NMDS. MRT and RDA tools were provided by the *mvpart* (MRT), *MVPARTwrap* (MRT) [28,29] and *vegan* (RDA) [27] packages.

## Results and discussion

### Temperature, carbon and nitrogen variations

#### Temperature

**Fig 1:**
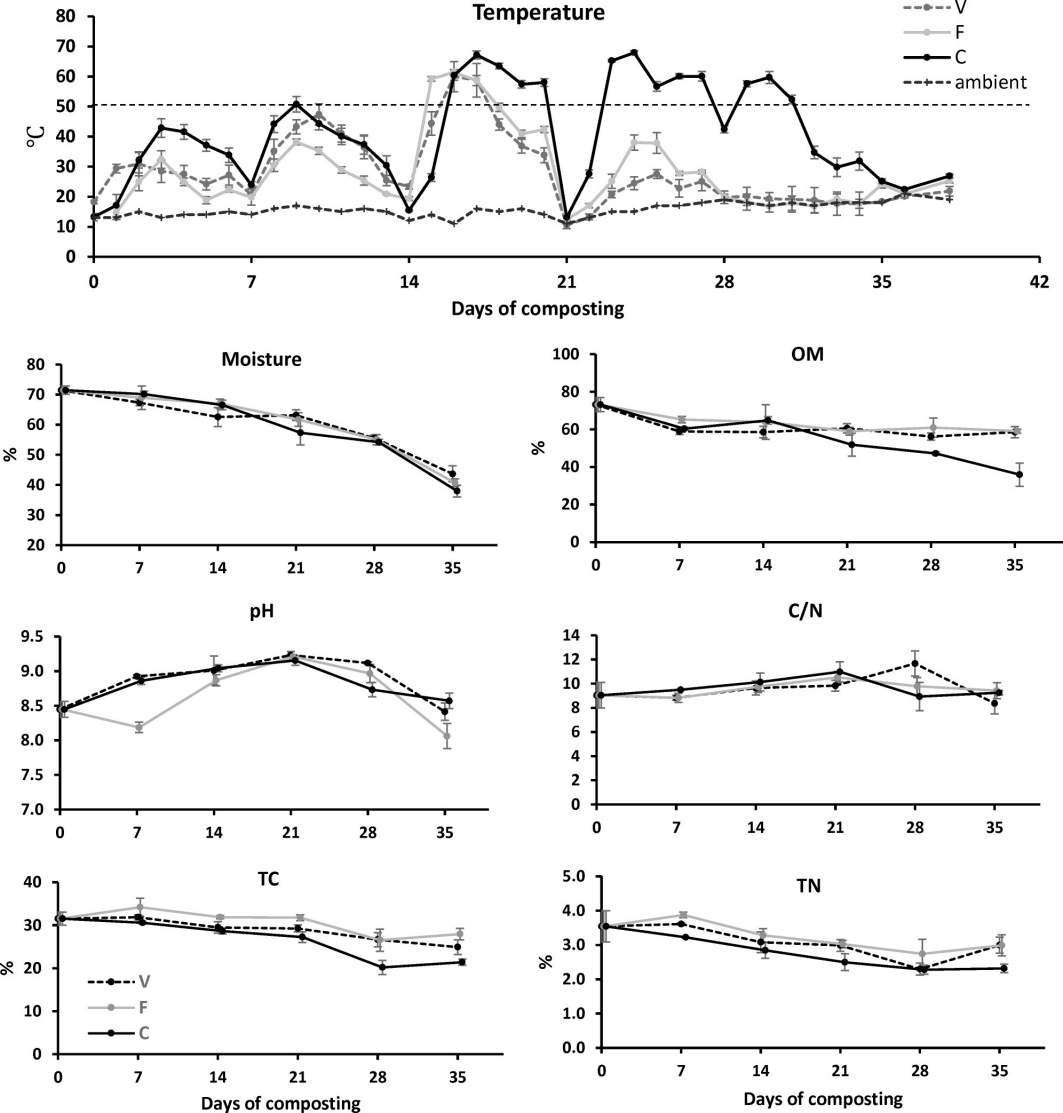
Temperature profile and moisture, organic matter, pH, C/N, total carbon and total nitrogen variation of V, F and C composts.

Temperature trends generally followed similar pattern among different amendments, with temperatures rapidly increasing after compost construction, peaking within 2–3 days, and declining subsequently, a pattern repeated after each turning (Fig 1). The total heating cycles lasted 4 wk for V and F composts and 5 wk for C compost, which were similar to composted poultry (initial pH 8.0 [30]) and cow (initial pH 7.4 [14]; initial pH 8.8 [11]) manure, suggesting that the NaOH/NaClO-contaminated poultry manure was successfully composted. The maximum temperature for V, F and C were 60.0, 61.4, and 67.9°C, respectively, and duration time of temperature > 55°C for V, F and C were 2, 4, and 12 d, respectively. Over 5 wk of composting, C compost achieved higher temperature (*p*< 0.001) than V and F. The greater heating of C compost may be attributed to a more favorable microbial community in the mature compost amendment. Other than the limitation of favorable microbial community, the V and F compost could also be influenced by the low initial C/N ratios, which may partially compromise the microbial development [2].

#### Moisture, OM and pH

The moisture content of poultry manure was 71.4% at construction and decreased gradually to 38.0%-43.7% after 35 d of composting (Fig 1) with no difference among V, F and C composts (*p* = 0.673). The OM content declined from 73.2% to 58.5% (V), 59.0% (F) and 35.9% (C) after 35 d (Fig 1). C compost had a lower (*p*<0.001) OM than V or F compost. This observation supports the higher composting temperatures observed for C, as composting temperature correlated with OM degradation. As the poultry manure used in the present study contained high sodium salt due to the regularly sanitization with NaOH and NaClO, the pH of poultry manure was approximately 8.45±0.12 at the beginning of composting, which was much higher than the poultry manure (6.4–8.3) used in other composting studies [2,5,30,31]. The pH value varied from 8.06 to 9.23 with no difference (*p* = 0.153) among the three composts. All pHs increased from D0 to D21, peaked on D21 at 9.23 (V), 9.21 (F) and 9.15 (C), and declined to 8.41 (V), 8.06 (F) and 8.57 (C) after 35 d (Fig 1). Except for slightly higher pH of the final compost product, these pH variations were comparable to two manure composting studies using more neutral manure. Zhong et al. [13] reported dairy manure composting initiated with pH 7.8±0.2 manure, ranged from 7.8–9.0, and reached a terminal pH of 8.0. Ren et al. [14] reported cow manure compost initiated with pH 7.4±0.1 manure, varied between 7.2–8.1, and reached a terminal pH of 7.6. Compared to another mixed poultry carcass-manure co-composting system [17], that the pH of the compost initiated at 7.7±0.1, but increased to 9.2±0.1 in mature compost, the present compost produced less alkaline product with more alkaline initial manure. The pH variation could be substantially affected by the nitrogen and carbon transformation during composting, that ammonification effect at the early stage of composting may partially lead the increase of compost pH and the degradation of lignocellulose carbon at late stage of composting may partly result in the compost pH decrease [2,18,32].

#### C/N, TC and TN

The C/N of all three composts ranged between 8.4–11.7 (Fig 1) with no difference among compost types (*p* = 0.971) or with composting time (*p* = 0.245). The compost TC and TN decreased continuously (Fig 1) over the composting period (*p*<0.001). Total C decreased faster in C than V compost, and V decomposed faster than F compost (*p*<0.001). For TN, C compost declined faster than V compost, which declined in a manner similar to F compost (*p*<0.001). The percent reduction in TC reached 11.4–32.1% in the present study, which was less than the 53% reduction observed in another aerobic poultry manure composting study [30], but comparable to the 19% reduction in a static poultry manure composting study [17]. Interestingly, lower TC reduction ratios were corresponded to higher alkaline conditions, as the pH mean values and high alkaline duration time were both higher in the present and static composting studies [17] than the aerobic compost study [30]. Since Chowdhury et al. [33] reported that co-composting bio-wastes with alkaline compounds enhanced carbon stabilization, the relatively low carbon loss in the present study may be highly related with the alkaline condition. The percent reduction in TN averaged at 20.9% in the present study, which was also low compared to the 50–80% reduction in the aerobic poultry manure composting study [30]. This could be partially attributed to the more lignocellulose amendments (40–60%) used in the aerobic composting study [30], that high lignocellulose content enhanced organic nitrogen degradation and the loss of gaseous nitrogen.

### Sodium, potassium, ammonia and EC variations

#### Extractable sodium

**Fig 2:**
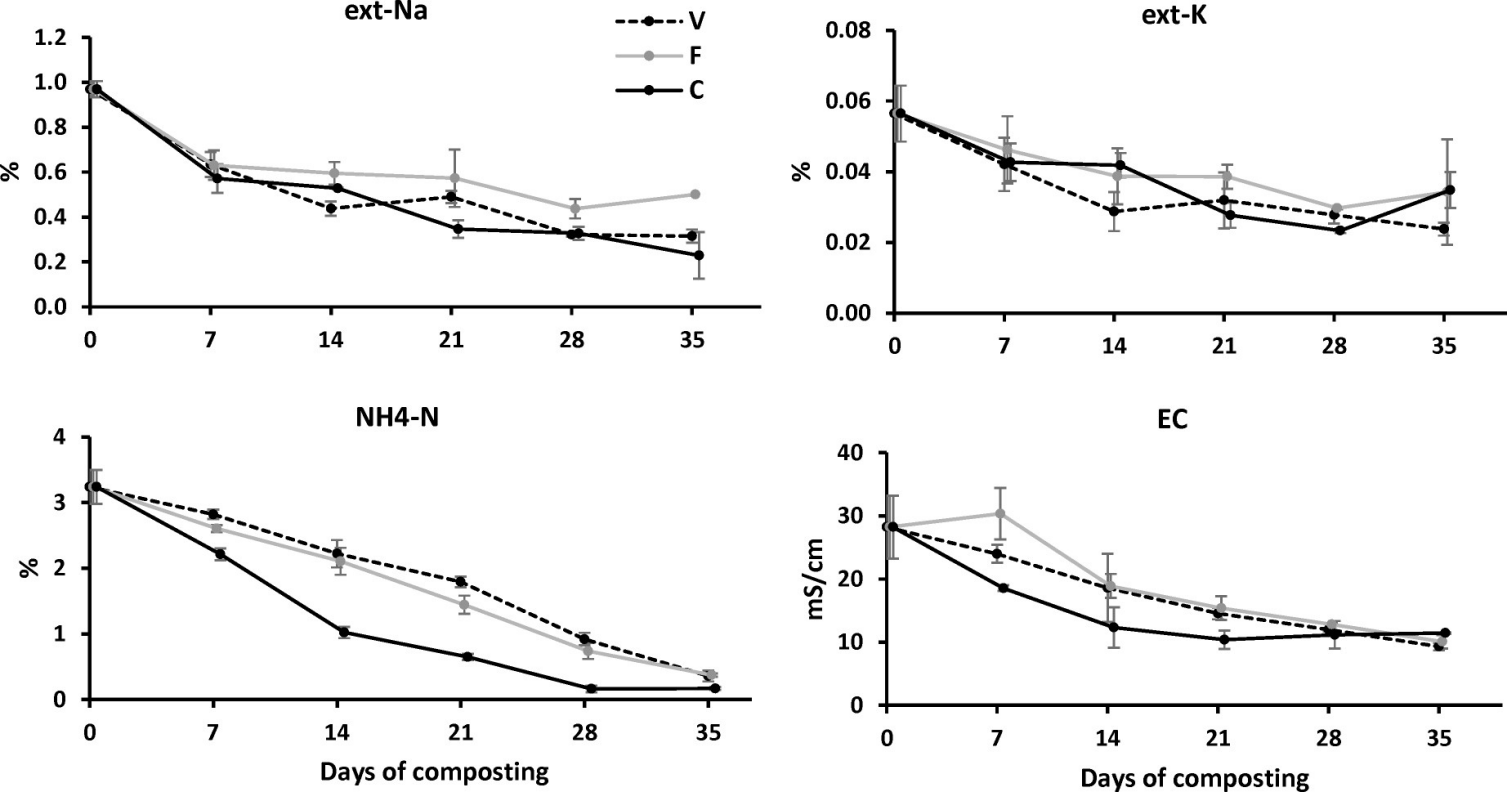
Extractable-Na, extractable-K, NH_4_-N and electrical conductivity variation of V, F and C composts.

Extractable-Na concentration rapidly declined in all compost types (*p*<0.001), with there being less (*p* = 0.007) ext-Na in compost C than F, which was similar to V compost (Fig 2). After 35 d of composting, the ext-Na concentration averaged at 0.31% (V), 0.50% (F) and 0.23% (C) in the three composts, meaning a 68.0% (V), 48.5% (F) and 76.3% (C) percent decrease in ext-Na. Due to regular usage of NaOH and NaClO sanitizer, the present compost initial Na concentration was 0.97±0.04%, which was much higher than most livestock and poultry manure (0.23–0.5%) [6,7] and sewage sludge (0.07–0.2%) [34,35], but comparable to food waste as it also has a high NaCl content. In a food waste and milk carton co-composting study, Brink [36] reported the Na concentration dropped from 0.88% to 0.29–0.37%, meaning 58.0–67.0% percent reduction after 4–8 mo composting. Lee et al. [37] reported the water-soluble Na concentration increased from 1.08% to 1.55% after 80 d of composting food waste amended with saw dust and paper mill sludge. Compared to the above studies, the present study resulted in comparable or higher ext-Na decrease rate with less composting time, which suggests the present composting strategy of alkaline poultry manure was efficient and practical in ext-Na immobilization. However, using food waste amendment (F) did result in a relatively high ext-Na concentration in the final compost. Using vegetable waste (V) or mature compost (C) as amendment could produce compost with lower ext-Na from poultry manure. Moreover, the ext-Na concentration in the final V (0.31%) and C (0.23%) compost was comparable to the poultry manure compost (0.20–0.34%) using relative neutral manure (pH 8.0±0.3) [30], which suggests V and C methods were reliable on ext-Na immobilization in the composting of NaOH/NaClO-contaminated poultry manure.

#### Extractable potassium and ammonia nitrogen

In contrast to the high concentration of ext-Na, the concentration of ext-K (Fig 2) was 10-times lower, decreasing (*p*<0.001) from 0.056% to 0.024–0.035% after 35 d with no difference among compost types (*p* = 0.189). The final ext-K concentration was generally lower than typical poultry manure compost of 0.65–0.87% ext-K [30] or food waste compost of 0.84–0.98% total-K [36]. The compost NH_4_-N (Fig 2) decreased rapidly and continuously after compost pile construction (*p*<0.001), a response similar to many other manure composting studies [11,14,30], and the decline in NH_4_-N was faster (*p*<0.001) in C than V and F compost.

#### Electrical conductivity

In accord with ext-Na variation, the compost EC (Fig 2) also decreased gradually over time (*p*<0.001), with compost C reduced faster than F and V compost (*p* = 0.020). The regular usage of NaOH and NaClO sanitizer not only resulted in elevated Na concentration, but also high EC values. EC values are generally 7–11 mS/cm for poultry manure [5,7], 1–9 mS/cm for cattle manure [5,7], and 2–3 mS/cm for green waste [2,15]. In contrast, the present alkaline poultry manure had an initial EC value of 28.2±5.0 mS/cm, which was much higher than most manure or green wastes. Thirty-five days of composting reduced EC from 28.2 mS/cm to 9.3 (V), 10.1 (F), and 11.4 (C) mS/cm, meaning 67.0%, 64.2% and 59.6% percent decrease, respectively. Compared to another poultry manure composting study [30], where the compost EC dropped from 18.7–21.4 mS/cm to 2.0–3.4 mS/cm, the present EC reduction level was less and the final EC values were high. However, compared to another food waste composting study [37], where compost EC increased from 39 to 48 mS/cm after 80 d of composting, the present treatment showed better EC reduction ability. The carbon amendment ratio and the initial EC in compost component could be important factors affecting compost EC, since the poultry manure study with 84.1–89.3% EC decrease [30] used higher ratio of agricultural green waste (40–60%) and lower EC of poultry manure than that used in the present study. Amendment properties may also influence compost EC, that F compost showed higher EC than V and C compost, which may result from the sea salt contained in the food waste amendment; and C compost showed lower EC than V and F compost, which may benefit from the mineral assimilation effect provided by the favorable microorganisms in the mature compost amendment. However, the decrease of compost EC could also be caused by mineral salt leaching. Unfortunately, the leaching effect was not investigated due to the very few leachate observed during composting. This is one of the limitations of the present study, further efforts should be carried out to study the corresponding microbial assimilation and leaching effect on the compost EC dynamics. As EC generally indicates the total salt content of the compost, as well as the agricultural quality for land application, EC value of 3–4 mS/cm has been considered as compost land-application limits [2,10]. The present composting study of NaOH/NaClO-contaminated poultry manure could be further optimized by additional steps to further reduce the EC.

### Correlations of chemical property

**Fig 3:**
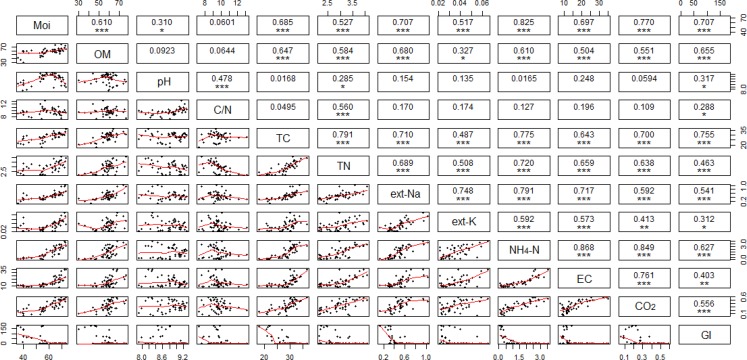
The correlation analyses of compost chemical and microbial properties over three composts. The diagonal shows the properties used for the correlation analyses; the lower triangle illustrates the x-y scatter plots of every two properties; and the upper triangle illustrates the correlation coefficient and *p* value of each correlation analysis. The *, **, and *** symbols represent significant levels of *p*<0.05, *p*<0.01, and *p*<0.001, respectively.

All the chemical properties were combined and analyzed for correlations regardless of compost type difference, which were summarized in Fig 3. The variation of ext-Na was significantly and linearly correlated with NH_4_-N (r = 0.791), ext-K (r = 0.748), EC (r = 0.717), TC (r = 0.710), and moisture (r = 0.707), meaning the decrease of ext-Na was consistent with the decline of NH_4_-N, ext-K, EC, TC and moisture (Fig 3). Other than ext-Na, NH_4_-N was another chemical property that had high correlations with most other chemical properties. NH_4_-N variation was significantly and linearly correlated with EC (r = 0.868), CO_2_ respiration rate (r = 0.849), moisture (r = 0.825), ext-Na (r = 0.791), TC (r = 0.775), and TN (r = 0.720), meaning the decrease of NH_4_-N was constant with the EC, CO_2_ respiration, moisture, ext-Na, TC and TN variations (Fig 3). Both ext-Na and NH_4_-N substantially contributed to the EC variation due to their relative high amount in the present poultry manure. The decrease of EC was correlated (*p*<0.001) with the decline of NH_4_-N and ext-Na, which further suggests that except for the common compost maturity indicator of NH_4_-N [38], EC and ext-Na could also be used as maturity and quality control indicators for NaOH/NaClO-contaminated poultry manure composting.

### Microbial properties and germination index

#### CO_2_ respiration rate and GI

**Fig 4:**
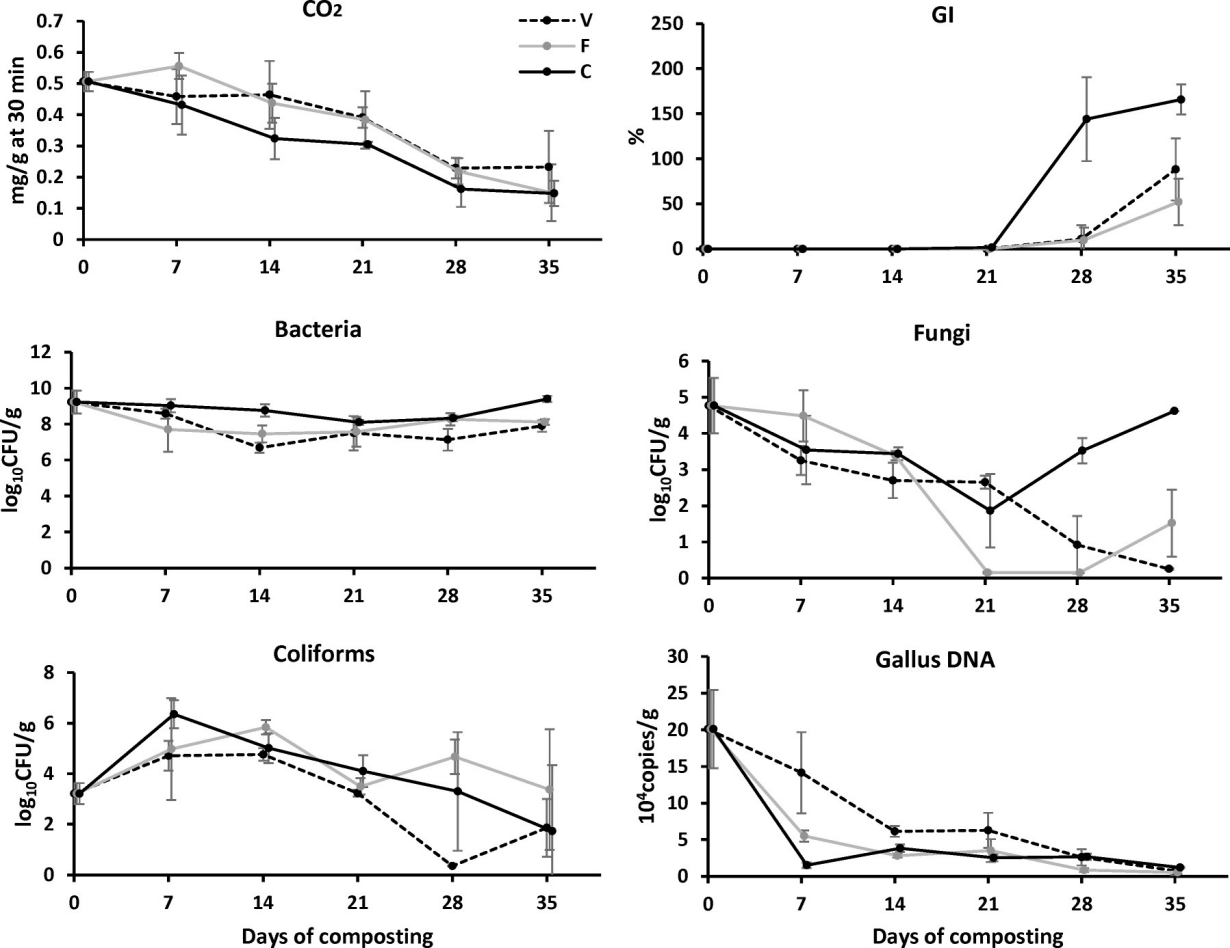
The variation of CO_2_ respiration rate, germination index, bacterial, fungal and coliform counts, and *Gallus-*specific DNA copy numbers over 35 d of composting.

The CO_2_ production rate decreased (*p*< 0.001) from 0.51 mg/g compost (after 30 min incubation in the detection chamber at room temperature) to 0.23 (V), 0.15 (F), and 0.15 (C) mg/g compost (Fig 4), which indicated the gradual reduction of the compost microbial activity. C compost exhibited a faster rate (*p* = 0.028) of CO_2_ reduction than V compost but similar to F compost. Regardless of the treatment difference, the decline of CO_2_ respiration was positively correlated (*p*<0.001, Fig 3) with the decrease of NH_4_-N (r = 0.849), moisture (r = 0.770), EC (r = 0.761) and TC (r = 0.700), which suggested the microbial CO_2_ respiration rate was simultaneously affected by the compost nitrogen (NH_4_-N), salinity (EC) and carbon (TC) nutrient conditions. The GI value remained at 0% over D0-21 for all three types of compost, and increased (*p*<0.001) to 88% (V), 52% (F) and 166% (C) after 35 d, respectively (Fig 4). C compost showed higher GI than V and F (*p*<0.001), suggested the lower phytotoxicity of C product. Compared to three other composting studies using less-alkaline poultry manure or agriculture green waste, where final GI values of compost product ranged around 16–64% [30], 38–122% [39], and 55–80% [15], respectively, the present treatment showed a high efficiency in the reduction of phytotoxicity in alkaline poultry manure.

#### Microbial numbers and DNA degradation

Compost bacterial numbers ranged from 7.1–9.4 log_10_ CFU/g over 35 d of composting (Fig 4), and C compost had higher (*p* = 0.004) bacterial numbers than F and V compost. Fungal numbers generally decreased for V and F compost over 35 d but only decreased over D0-21 and increased over D21-35 for C compost with fungal numbers being higher than V and F compost (*p* = 0.002). The compost fungal numbers ranged from 0–4.8 log_10_ CFU/g, which was less than the bacterial numbers of 3.2–8.1 log_10_ CFU/g, meaning at least 10^3^ times less fungi than bacteria in compost. The substantial higher numbers of bacteria than fungi agreed with other composting studies [18,40,41] and further suggested that the bacterial community played more important role during composting than fungi. The total coliforms generally increased over D0-14 and decreased from D14 to 35, and finally reduced to 1.9 (V), 3.4 (F) and 1.7 (C) log_10_ CFU/g on D35 (Fig 4), which generally meet the China national organic fertilizer requirement (NY884-2004) of less than 2.0 log_10_ CFU/g. The *Gallus*.*gallus* (chicken) specific 12S rRNA gene was reduced (*p*<0.001) from 20.1 ×10^4^ copies/g to 0.8 (V), 0.5 (F) and 1.2 (C) ×10^4^ copies/g after 35 d of composting (Fig 4), representing 96%, 97% and 94% percent decrease, respectively, with no difference among compost types (*p* = 0.066). The animal-specific DNA degraded faster in the present manure compost (94–97% percent decrease after 35 d) than in previous cow carcass compost (77–88% percent decrease after 207–267 d) [22,32], partially because there was no carcass in the present manure compost.

### Bacterial diversity and relationship with chemical properties

#### Bacterial composition and dynamics

**Fig 5:**
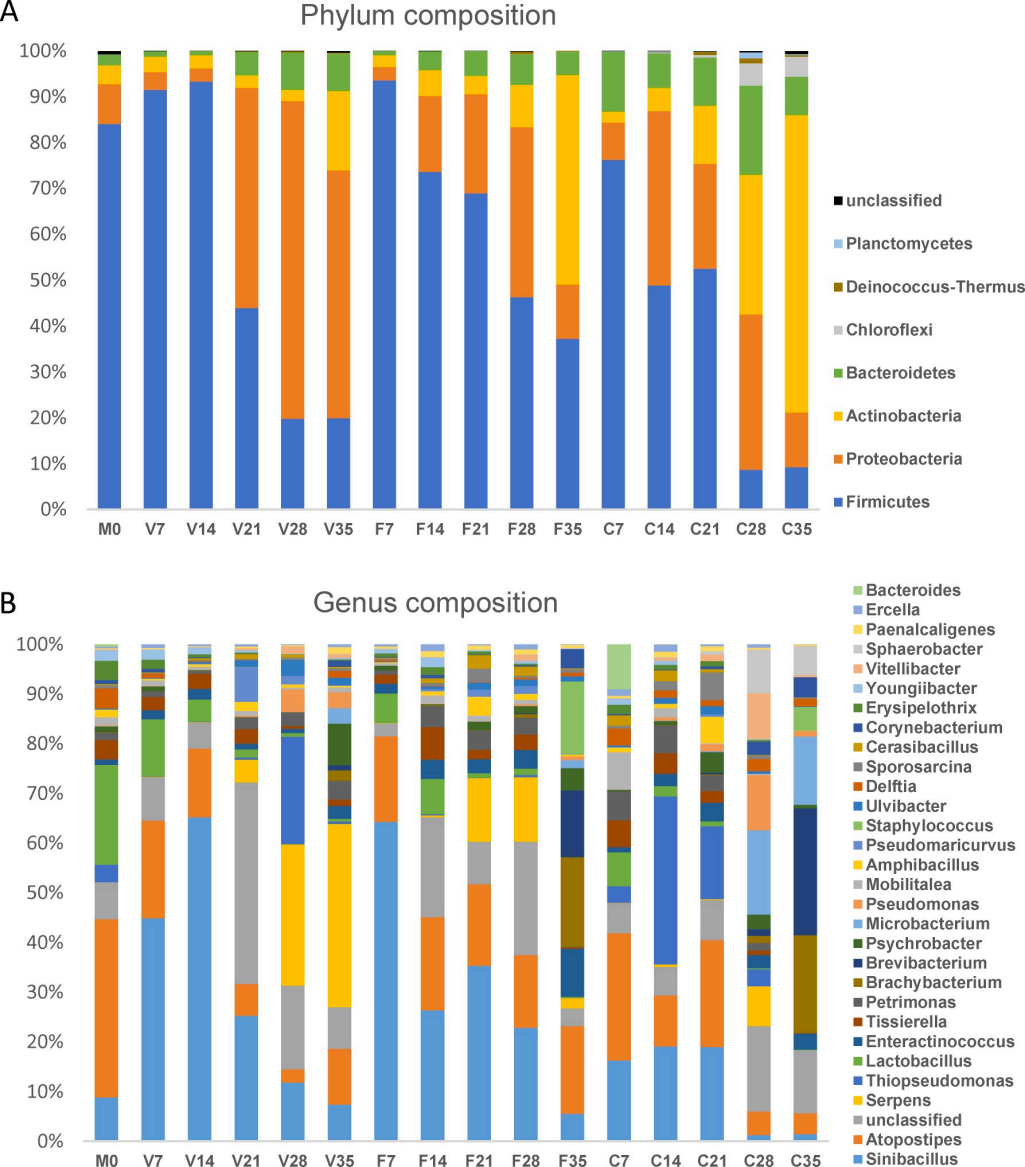
The dynamics of compost bacterial phylum and genus composition of V, F and C composts. M0, D0 poultry manure; 0, 7, 14, 21, 28, 35, sampling time points. The species legend from bottom to top was illustrated according to the corresponding colors in each column.

The phylum and genus of compost bacteria determined by the Illumina MiSeq sequencing method is summarized in Fig 5. A total of 17 phyla were identified over 35 d of composting, and eight phyla (including one unclassified group) showing an average abundance level more than 0.1%, were further analyzed for compost phylum composition and variations (Fig 5A). Overall, Firmicutes, Proteobacteria, Actinobacteria, and Bacteroidetes were the four predominant bacteria phyla identified with the abundance ratio averaged at 54.2%, 24.4, 13.5% and 6.7%, respectively, and accounted for 92.1–99.9% phyla at different compost times in total. The bacterial phylum composition was generally similar among different treatments (*p* = 0.431) revealed by ANOSIM analysis, whereas it became progressively diverged over composting period (*p* = 0.009) with the dominant bacterial phyla changing from Firmicutes to Proteobacteria, Actinobacteria, and Bacteroidetes (Fig 5A). At the genus level, a total of 452 genera were identified, whereas 30 genera (including one unclassified group) showed an average abundance ratio higher than 0.5%, which were further extracted and analyzed for genus composition (Fig 5B). Overall, nine genus taxa showed average abundance ratio higher than 2.0%, which were *Sinibacillus* (Firmicutes, 20.8%), *Atopostipes* (Firmicutes, 12.6%), unclassified (10.0%), *Serpens* (Proteobacteria, 5.5%), *Thiopseudomonas* (Proteobacteria, 4.4%), *Lactobacillus* (Firmicutes, 3.4%), *Enteractinococcus* (Actinobacteria, 2.3%), *Tissierella* (Firmicutes, 2.2%), and *Petrimonas* (Bacteroidetes, 2.1%), respectively. Consistent with the phylum structure, the genus composition was similar among the three treatments (*p* = 0.345), but changed profoundly and progressively over composting period (*p* = 0.003).

Compost bacterial community structure could be affected by the compost material and sequencing methods substantially. Based on the Illumina Miseq next-generation sequencing technology, the present bacterial community generally showed high phyla but low genera similarity compared to other composting studies. In poultry manure (initial pH 7.6–7.8) and poultry carcass co-composting study, Wang et al. [17] showed the top four dominant phyla were Firmicutes, Proteobacteria, Actinobacteria, and Bacteroidetes, respectively, a finding confirmed by the present study. However, the bacterial genera were less identified (<50%) in that study, and *Corynebacterium*, *Virgibacillus*, *and Bacteroides* were the relatively high abundance genera. More genera (>90%) were identified in the present study, with *Corynebacterium*, *Virgibacillus*, *and Bacteroides* as low abundance (0.1–0.7%) genera. These substantial differences could be caused by not only the compost component difference, but also the NGS method difference of 16S targeting region, as Wang et al. [17] sequenced V3-V4 region of 16S rRNA gene in contrast to the present study of V6-V8 region. Thus, both the poultry manure initial property and NGS sequencing method could have influenced the bacterial genera composition analysis.

In cow manure composting studies, the dominant phyla were Proteobacteria, Bacteroidetes, Firmicutes, Actinobacteria, and Chloroflexi [13]; Firmicutes, Proteobacteria, Actinobacteria, and Bacteroidetes [42]; Bacteroidetes and Proteobacteria [14] or Bacteroidetes, Gammaproteobacteria, Alphaproteobacteria, Actinobacteria, and Firmicutes [11]. The present study showed similar phylum composition compared to the above cow manure composting studies, but a different genus structure, that high abundance genera in the cow manure compost of *Calditerricola* (Firmicutes), *Cellvibrio* (Proteobacteria), *Thermobifida* (Actinobacteria), and *Chryseolinea* (Bacteroidetes) were seldom found in the present study. Compared to agriculture green waste composting studies, the dominant phyla of Actinobacteria, Firmicutes, Proteobacteria, and Bacteroidetes were similar to those of the present study, with the exception of Chloroflexi and Gemmatimonadetes phyla. And the dominant genera of *Thermobifida*, *Thermopolyspora*, *Clostridium*, *Ureibacillus*, and *Lysinibacillus* [12,15] were all less than 0.1% abundance in the present study. Both cow manure and green waste compost had higher proportion of lignocellulose components, which may require bacterial genera with higher cellulose and lignin enzymatic degradation ability compared to the chicken manure used in the present study. Thus, agricultural manure and green waste compost with different initial component may be consistent in the bacterial phylum composition, but significantly different and better characterized by their genus structures and variations.

#### Compost classification and chemical property impact on bacterial dynamics

**Fig 6:**
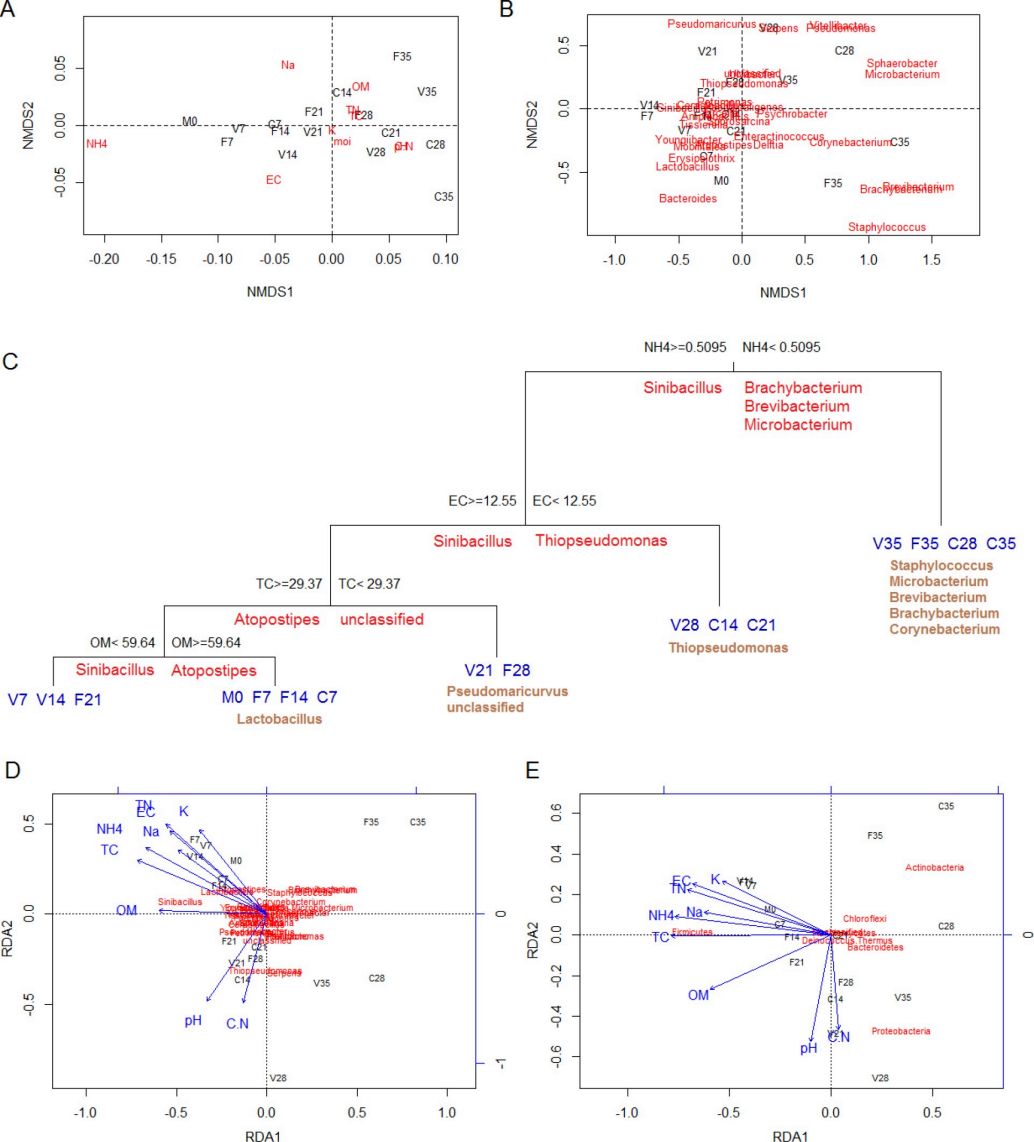
Classification analyses of compost samples using unconstrained and constrained clustering methods. A, NMDS analysis based on chemical properties; B, NMDS analysis based on bacterial genus composition; C, MRT analysis based on chemical properties and bacterial genus composition; D, RDA analysis based on compost chemical properties and bacterial genus composition; E, RDA analysis based on compost chemical properties and bacterial phylum diversity. M0, D0 poultry manure; 0, 7, 14, 21, 28, 35, sampling time points.

Based on the chemical property and bacterial genera, the samples progressively collected from the compost were classified with NMDS analysis as shown in Fig 6A and 6B, respectively. As shown in Fig 6A, the chemical dissimilarity of samples compared to D0 manure (M0) increased progressively according to the compost types and time points, with samples on D35 (V35, F35, C35) distinguished the most from D0 and among compost types, which indicated the maturing process as well as the profound differences in the three composts at maturity. According to the distance of deviance to the ordination center, NH_4_-N, EC and ext-Na were the primary contributors to the sample classification, meaning the top three indicators of compost progressive maturity based on chemical changes. As shown in Fig 6B, the dissimilarity of the bacterial genera also increased with the composting time, with D28 and D35 samples showing the farthest distance (highest dissimilarity) to the D0 samples within each treatment. The genera *Staphylococcus* (Firmicutes), *Brevibacterium* (Actinobacteria), and *Brachybacterium* (Actinobacteria) were the top three contributors to the sample classification.

Different from NMDS analysis, the MRT and RDA analyses were constrained clustering method, that classified compost samples based on both chemical properties and bacterial diversities. In the MRT analysis (Fig 6C), combining the measured chemical properties (OM, pH, TC, TN, C/N, ext-Na, ext-K, NH_4_-N and EC) and 30 bacteria genera identified by Illumina MiSeq sequencing, the main factors that affected the bacterial community variations were ranked as NH_4_-N, EC, TC and OM. The NH_4_-N content differentiated the compost into the two groups of early-middle (NH_4_-N>0.5095%) and late (NH_4_-N<0.5095%) stages, the EC content split the remaining compost into the early (EC>12.55 mS/cm) and middle (EC<12.55 mS/cm) stages, while the TC and OM discriminated the remaining compost into several subgroups of early stage. The total tree splits explained 52.5% of the variation in bacterial genera, with NH_4_-N, EC, TC and OM explaining 25.7%, 13.4%, 8.5% and 4.9%, respectively. The relatively low explanation ratio was partially caused by the complexity of the bacterial genus community as well as the minimum variation of large proportions of genera as indicated in the ordination center of NMDS analysis (Fig 6B). Considering the indicator species of tree nodes and leaves, the discriminant genera for different stages of composting process were *Sinibacillus*, *Atopostipes*, *Lactobacillus* and *Pseudomaricurvus* for the early stage of composting, *Thiopseudomonas* for the middle stage of composting, and *Staphylococcus*, *Microbacterium*, *Brevbacterium*, *Brachybacterium* and *Corynebacterium* for the late stage of composting. In the RDA analysis, combining the measured chemical properties and either 30 bacterial genera (Fig 6D) or eight bacterial phyla (Fig 6E), the significant chemical factors that affected bacteria variations ranked as NH_4_-N, TC, TN and EC for both genus and phylum community analyses. The discriminate genera were *Sinibacillus* (Firmicutes), *Thiopseudomonas* (Proteobacteria)_,_ and *Serpens* (Proteobacteria) (Fig 6D), and the discriminate phyla were Firmicutes, Proteobacteria, and Actinobacteria (Fig 6E).

Interestingly, ext-Na was the discriminate chemical factor in NMDS analysis but not in MRT or RDA analyses, which suggests ext-Na indicated compost maturity but did not influence bacterial dynamic substantially. Since the decrease of ext-Na could be highly contributed by the microbial immobilization activity, the variation of ext-Na did not drive the bacterial community changing, which suggests that the inhibition effect caused by ext-Na could be low. In contrast to ext-Na, properties of NH_4_-N and EC were both significant factors in chemical maturity classification (NMDS, Fig 6A) and chemical-bacteria correlation analyses (MRT and RDA, Fig 6D and 6E). Ammonia nitrogen and salt (indicated by EC) had significant effects on the compost maturity and bacterial dynamics, which agreed with the previous reports that the concentration and component of nitrogen affected the microbial community [38,43]. In contrast to ext-Na, NH_4_-N and EC, property of TC was only noted significant in the chemical-bacterial correlation analysis of MRT and RDA, but not in the chemical classification analysis (NMDS), which indicated that although the TC conditions changed to a small extent during composting, the effect of TC on bacterial community variation was significant, as organic carbon is an essential nutrient element for bacterial growth and activity [12,38].

Although 30 bacterial genera were identified with abundance averaging higher than 0.5%, only a few genera (*Sinibacillus*, *Atopostipes*, *Lactobacillus*, *Thiopseudomonnas*, *Pseudomaricurvus*) were discriminate species for the early and middle stage of compost as illustrated by MRT analysis (Fig 6C), implying these species may play important roles in carbon, nitrogen and sodium metabolism or degradation. *Sinibacillus* (Bacillaceae 2, Bacillales, Bacilli, Firmicutes) [44] was the predominant and discriminate genus over the first 21 d of composting. *Sinibacillus* has been characterized by optimal growth at 50–63°C, pH 8.0, 1–1.5% (w/v) NaCl, and is capable of using either organic nitrogen, (NH_4_)_2_SO_4_ or NH_4_Cl as the sole nitrogen sources. These properties support the findings that *Sinibacillus* survived and became predominant in the early stage of composting, i.e. under thermophilic, alkaline, high ext-Na, and high NH_4_-N conditions. The *Atopostipes* and *Lactobacillus* genera were less reported in previous compost studies, probably due to their lower tolerance to compost thermophilic temperatures [45,46]. In contrast, *Pseudomaricurvus* and *Thiopseudomonnas* have been noted in the middle stage compost of other composting studies, due to their properties of polysaccharide and lignocellulosic degradation and tolerance to high salinity [47,48]. Further efforts will be conducted to isolate these specific bacterial genera, investigate their metabolism and evaluate their potential usage as compost inocula.

## Conclusions

Co-composting of NaOH/NaClO-contaminated poultry manure with mature compost and vegetable waste was more efficient at lowering ext-Na and EC than food waste. Co-composting with mature compost resulted in the highest compost temperatures (*p*<0.001) and greatest reduction in OM, TC, TN and NH_4_-N (*p*<0.05), therefore is more desirable than vegetable and food waste. Maturity was primarily indicated by NH_4_-N, EC and ext-Na. Bacterial dynamics was profoundly influenced by NH_4_-N, EC and TC, with the respect decrease resulted in discriminate genera shift from *Sinibacillus*, *Thiopseudomonas* to *Brevbacterium*, *Brachybacterium*, and *Microbacterium*. The ext-Na decrease indicated compost maturity but did not influence bacterial dynamics.
